# Supplementary material for: MAIT cells regulate NK cell-mediated tumor immunity
Source: Nat Commun. 2021 Aug 6;12:4746. doi: 10.1038/s41467-021-25009-4 (PMC8346465; doi:10.1038/s41467-021-25009-4)
Supplement: Supplementary file 1 — Supplementary Information [file 41467_2021_25009_MOESM1_ESM.pdf]

MAIT cells regulate NK cell-mediated tumor immunity

Supplementary information.

Emma V. Petley[1,2], Hui-Fern Koay[3,4], Melissa A. Henderson[1,2], Kevin Sek[1,2], Kirsten L. Todd[1,2], Simon P. Keam[1,2,5], Junyun Lai[1,2], Imran G. House[1,2], Jasmine Li[1,2],Magnus Zethoven[2,6], Amanda X. Y. Chen[1,2], Amanda J. Oliver[1,2], Jessica Michie[1,2], Andrew J Freeman[1,2], Lauren Giuffrida[1,2], Jack D. Chan[1,2], Angela Pizzolla[1,2], Jeffrey Y. W. Mak[7,8], Timothy R. McCulloch[9], Fernando Souza-Fonseca-Guimaraes[9], Conor J. Kearney[1,2], Rosemary Millen[1,2], Robert G. Ramsay[1,2], Nicholas D. Huntington[10-12], James McCluskey[3], Jane Oliaro[1,2,13], David P. Fairlie[7,8], Paul J. Neeson[1,2], Dale I. Godfrey[3,4,15], Paul A. Beavis[1,2,14,15], Phillip K. Darcy[1,2,12,14,15]

[1]Cancer Immunology Program, Peter MacCallum Cancer Centre, Melbourne, VIC, Australia.
[2]Sir Peter MacCallum Department of Oncology, The University of Melbourne, Parkville, VIC, Australia.
[3]Department of Microbiology & Immunology, Peter Doherty Institute for Infection and Immunity, University of Melbourne, Melbourne, VIC, Australia.
[4]Australian Research Council Centre of Excellence in Advanced Molecular Imaging, University of Melbourne, Melbourne, VIC, Australia.
[5]Tumour Suppression and Cancer Sex Disparity Laboratory, Peter MacCallum Cancer Centre, Melbourne, VIC, Australia.
[6]Bioinformatics Core Facility, Peter MacCallum Cancer Centre, Melbourne, VIC, Australia.
[7]Institute for Molecular Bioscience, The University of Queensland, Brisbane, QLD, Australia.
[8]Australian Research Council Centre of Excellence in Advanced Molecular Imaging, The University of Queensland, Brisbane, QLD, Australia.
[9]University of Queensland Diamantina Institute, The University of Queensland, Brisbane, QLD, Australia.
[10]Department of Medical Biology, Faculty of Medicine, Dentistry and Health Sciences, University of Melbourne, Melbourne, VIC, Australia.
[11]Division of Molecular Immunology, Walter and Eliza Hall Institute of Medical Research, Melbourne, VIC, Australia.
[12]Biomedicine Discovery Institute and the Department of Biochemistry and Molecular Biology, Monash University, Melbourne, VIC, Australia.
[13]Department of Immunology, Monash University, Melbourne, VIC, Australia.
[14]Department of Pathology, University of Melbourne, Melbourne, VIC, Australia.
[15]These authors jointly supervised this work.

Supplementary Figure 1

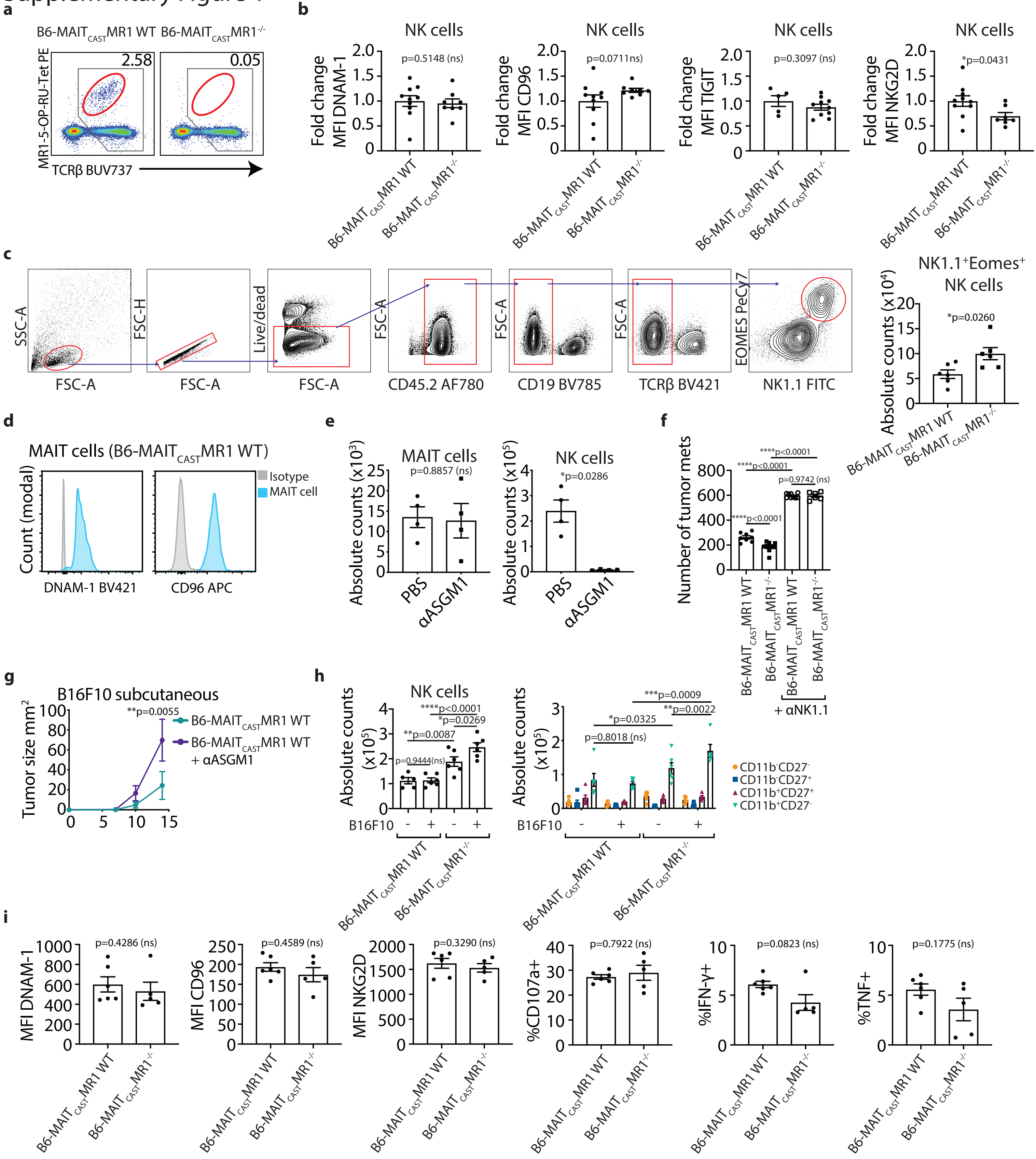

Supplementary Fig. 1 Immune cell phenotype in B6-MAIT$_{cast}$MR1 WT and B6-MAIT$_{cast}$MR1$^{-/-}$ mice. **(a)** Representative flow cytometry plots of MAIT (CD45.2^{+}CD19^{-}TCRβ^{+}MR1-5-OP-RU-tetramer^{+}) cells in the lungs of indicated mice. **(b)** Expression of DNAM-1, CD96, TIGIT and NKG2D on NK cells from lungs of indicated mice. Data is presented as mean ± SEM of n = 10 (B6-MAIT$_{cast}$MR1 WT, DNAM-1, CD96, NKG2D), n = 8 (B6-MAIT$_{cast}$MR1$^{-/-}$, DNAM-1, CD96), n = 7 (B6-MAIT$_{cast}$MR1$^{-/-}$, NKG2D), n = 5 (B6-MAIT$_{cast}$MR1 WT, TIGIT) and n = 10 (B6-MAIT$_{cast}$MR1$^{-/-}$, TIGIT) independent mice from two combined experiments, two-tailed Mann-Whitney test. **(c)** Gating strategy of NK cells (CD45.2^{+}CD19^{-}TCRβ^{-}NK1.1^{+}Eomes^{+}) and number of NK cells in B6-MAIT$_{cast}$MR1 WT and B6-MAIT$_{cast}$MR1$^{-/-}$ mice. Data is presented as mean ± SEM of n = 6 independent mice from one independent experiment, two-tailed Mann-Whitney test. **(d)** Representative histograms of expression of DNAM-1 and CD96 on MAIT cells from the lungs of B6-MAIT$_{cast}$MR1 WT mice. Data represents two independent experiments. **(e)** B6-MAIT$_{cast}$MR1 WT mice were treated with anti-asialo GM-1 (αASGM1) on days 0 and 1 and lungs were harvested day 7. The number of MAIT and NK cells in the lungs were assessed by flow cytometry. Data is presented as mean ± SEM of n = 4 independent mice from one independent experiment, two-tailed Mann-Whitney test. **(f)** 2 x10^{5} B16F10 tumour cells were injected i.v. into B6-MAIT$_{cast}$MR1 WT mice or B6-MAIT$_{cast}$MR1$^{-/-}$ mice and NK cells were targeted with αNK1.1 on days -3, 0 and 3. Lungs were harvested and the number of tumor metastases enumerated 14 days after inoculation. Data presented as mean ± SEM of n = 8 (non-treated B6-MAIT$_{cast}$MR1 WT), n = 9 (non-treated B6-MAIT$_{cast}$MR1$^{-/-}$ and αNK1.1 treated B6-MAIT$_{cast}$MR1 WT) and n = 6 (αNK1.1 treated B6-MAIT$_{cast}$MR1$^{-/-}$) independent mice from one independent experiment, One-way ANOVA. This experiment was run concurrently with **Figure 1f** and PBS controls are the same data points. **(g)** 3 x 10^{4} B16F10 tumor cells were injected s.c. into B6-MAIT$_{cast}$MR1 WT mice and NK cells targeted via treatment with αASGM1 on days -1 and 0. Data is presented as mean ± SEM of n = 6 independent mice from a representative of two independent experiments, Two-way ANOVA. **(h-i)** B6-MAIT$_{cast}$MR1 WT and B6-MAIT$_{cast}$MR1$^{-/-}$ mice received 2 x 10^{5} B16F10 tumor cells i.v. and lungs were harvested at day 5. **(h)** NK cell numbers and maturation status was determined by flow cytometry. Data is presented as mean ± SEM of n = 5 (B6-MAIT$_{cast}$MR1 WT) and n = 6 (B6-MAIT$_{cast}$MR1 WT + B16F10, B6-MAIT$_{cast}$MR1$^{-/-}$ and B6-MAIT$_{cast}$MR1$^{-/-}$ + B16F10) independent mice from one independent experiment, One-way ANOVA (left). Data is presented as mean ± SEM of n = 6 (B6-MAIT$_{cast}$MR1 WT, B6-MAIT$_{cast}$MR1 WT + B16F10, B6-MAIT$_{cast}$MR1$^{-/-}$) and n = 5 (B6-MAIT$_{cast}$MR1$^{-/-}$ + B16F10) independent mice from one independent experiment, Two-way ANOVA (right). **(i)** NK receptor expression, CD107a expression and cytokine production from NK cells was assessed via flow cytometry. Data is presented as mean ± SEM of n = 6 (B6-MAIT$_{cast}$MR1 WT) and n = 5 (B6-MAIT$_{cast}$MR1$^{-/-}$) independent mice from one independent experiment, two-tailed Mann-Whitney test. *p < 0.05, **p < 0.01, ***p < 0.001, ns = non-significant.

Supplementary Figure 2

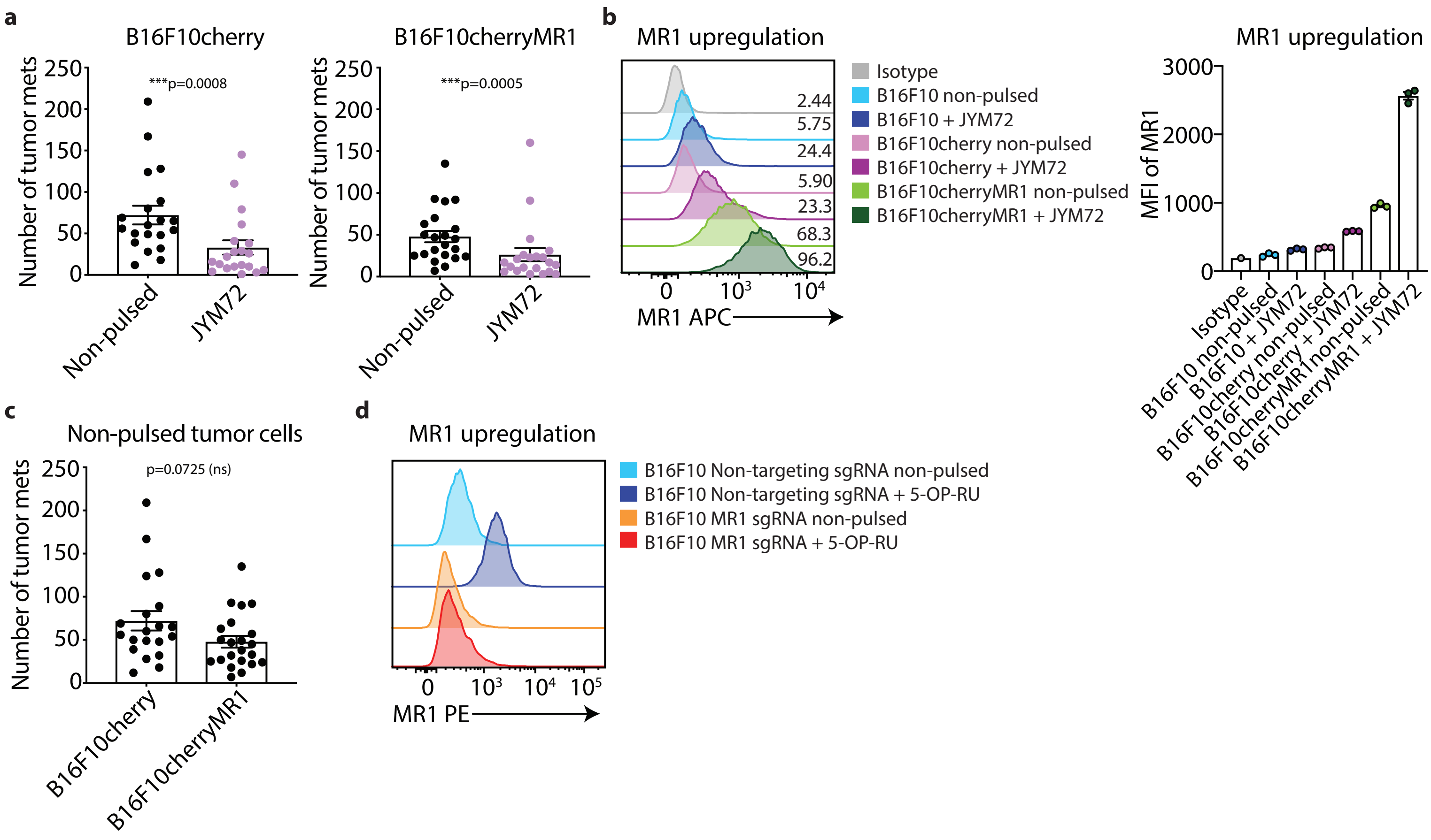

Supplementary Fig. 2 Modulation of B16F10 metastasis by presentation of MAIT cell antigens and MR1 expression. B16F10 cells were transduced with retrovirus encoding Cherry MR1 or a control (Cherry) vector. **(a)** Tumor cells were pulsed with 10 μM of JYM72 for 4 hours at 37°C and 2 x 10^5 cells were injected into C57BL/6 mice. Tumor metastases were enumerated 14 days after inoculation. Data is presented as mean ± SEM of $n = 20$ (non-pulsed B16F10cherry and JYM72 pulsed B16F10cherry), $n = 22$ (non-pulsed B16F10cherryMR1) and $n = 21$ (JYM72 pulsed B16F10cherryMR1) independent mice from three combined experiments, two-tailed Mann-Whitney test. **(b)** Representative histograms and bar graph of MR1 on B16F10 parental cells, B16F10cherry and B16F10cherryMR1 cells as determined by flow cytometry following incubation with or without 10 μM JYM72 for 4 hours at 37°C. Data is presented as mean ± SEM of triplicate cultures and represents three independent experiments. **(c)** Data shows non-pulsed tumor groups from **a**. two-tailed Mann-Whitney test. **(d)** Representative histograms of MR1 expression on B16F10 non-targeting sgRNA and B16F10 MR1 sgRNA cells as determined by flow cytometry following incubation with or without 10 μM 5-OP-RU for 4 hours at 37°C. ***$p < 0.001$, ns = non-significant.

Supplementary Figure 3

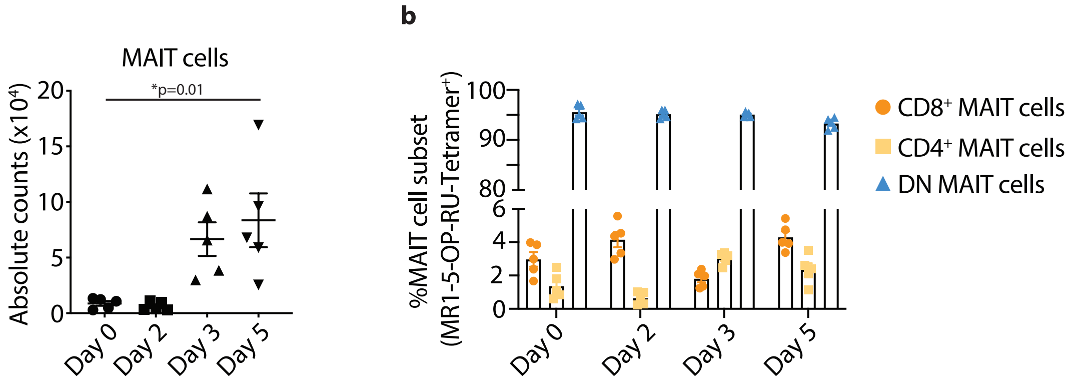

Supplementary Fig. 3 Time course of effect of intranasal 5-OP-RU treatment on MAIT cells. B6-MAIT$_{cast}$MR1 WT mice were treated with PBS or 5-OP-RU as per **Figure 3a**. At indicated days mice were euthanized and lung immune cells analyzed by flow cytometry. **(a)** Absolute number of MAIT cells in the lungs of mice following intranasal administration of 5-OP-RU at indicated time points. Data is presented as mean ± SEM of $n = 5$ independent mice from one independent experiment, One-way ANOVA. **(b)** Proportions of CD8$^+$, CD4$^+$ or double negative (DN) MAIT cells at indicated time points. Data is presented as mean ± SEM of $n = 5$ independent mice from 1 independent experiment. $*p < 0.05$.

Supplementary Figure 4

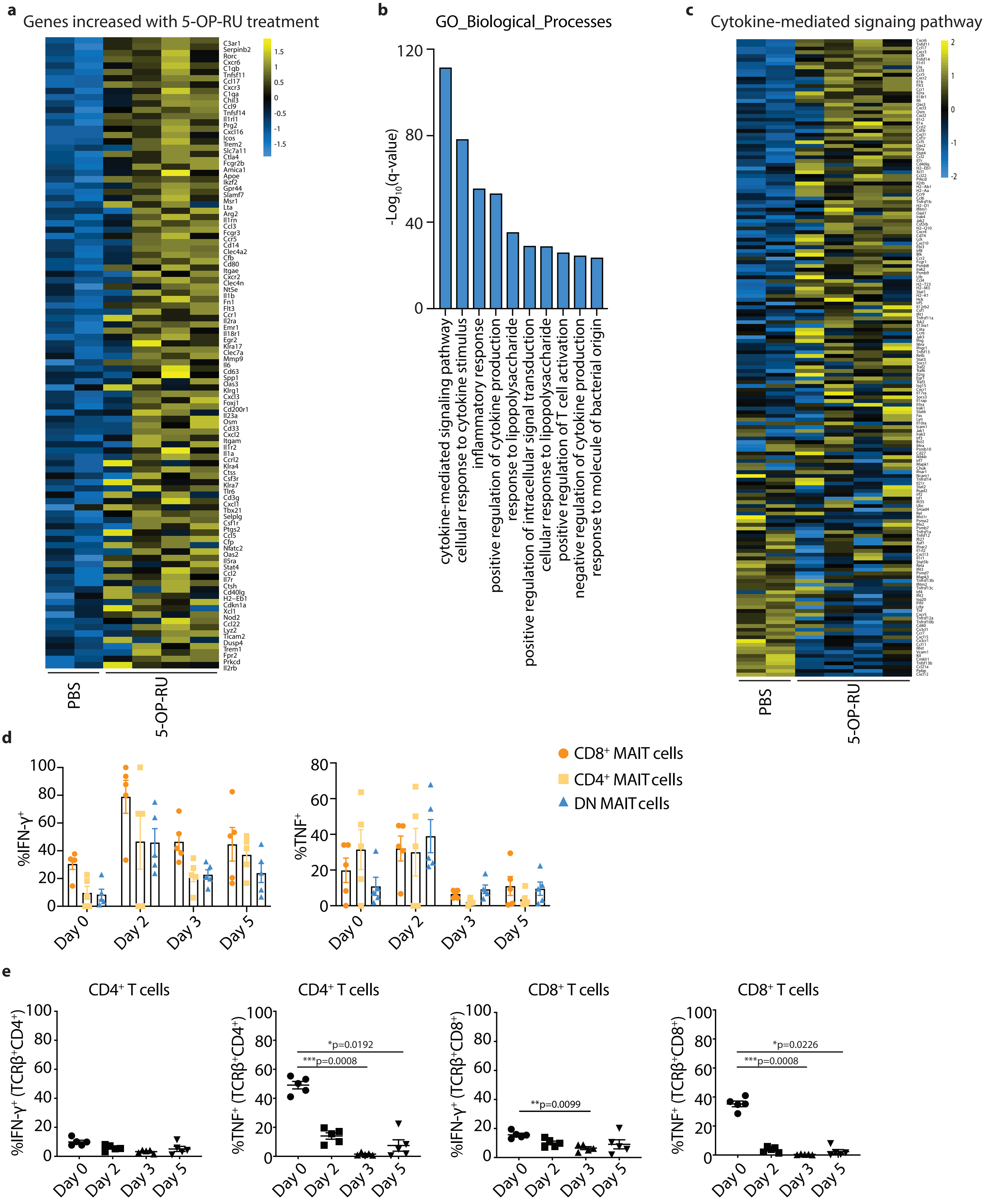

Supplementary Fig. 4 Intranasal 5-OP-RU treatment modifies gene expression in lung tissue. (a) Gene expression analysis of whole lung tissue five days after intranasal 5-OP-RU treatment in B6-MAIT$_{cast}$MR1 WT mice as in **Figure 3a**. Genes ranked according to fold increase after 5-OP-RU treatment. Top 100 differentially expressed genes are shown, excluding genes not detected in PBS condition. **(b)** Gene set analysis of differentially expressed genes (5-OP-RU versus. PBS) against GO biological processes. **(c)** Heat map of gene expression of the cytokine-mediated signaling pathway identified from GO biological processes. Data is represented as the gene expression identified from individual mice in indicated treatment groups n = 2 (PBS) or n = 4 (5-OP-RU). **(d-e)** B6-MAIT$_{cast}$MR1 WT mice were treated with PBS or 5-OP-RU as per **Figure 3a**. Production of IFN-γ and TNF by MAIT cell subsets (**d**) CD4$^+$ and CD8$^+$ T cell (**e**) at indicated time points. Data is presented as mean ± SEM of n = 5 independent mice from one independent experiment, Kruskal-Wallis test. *p < 0.05, **p < 0.01, ***p < 0.001.

Supplementary Figure 5

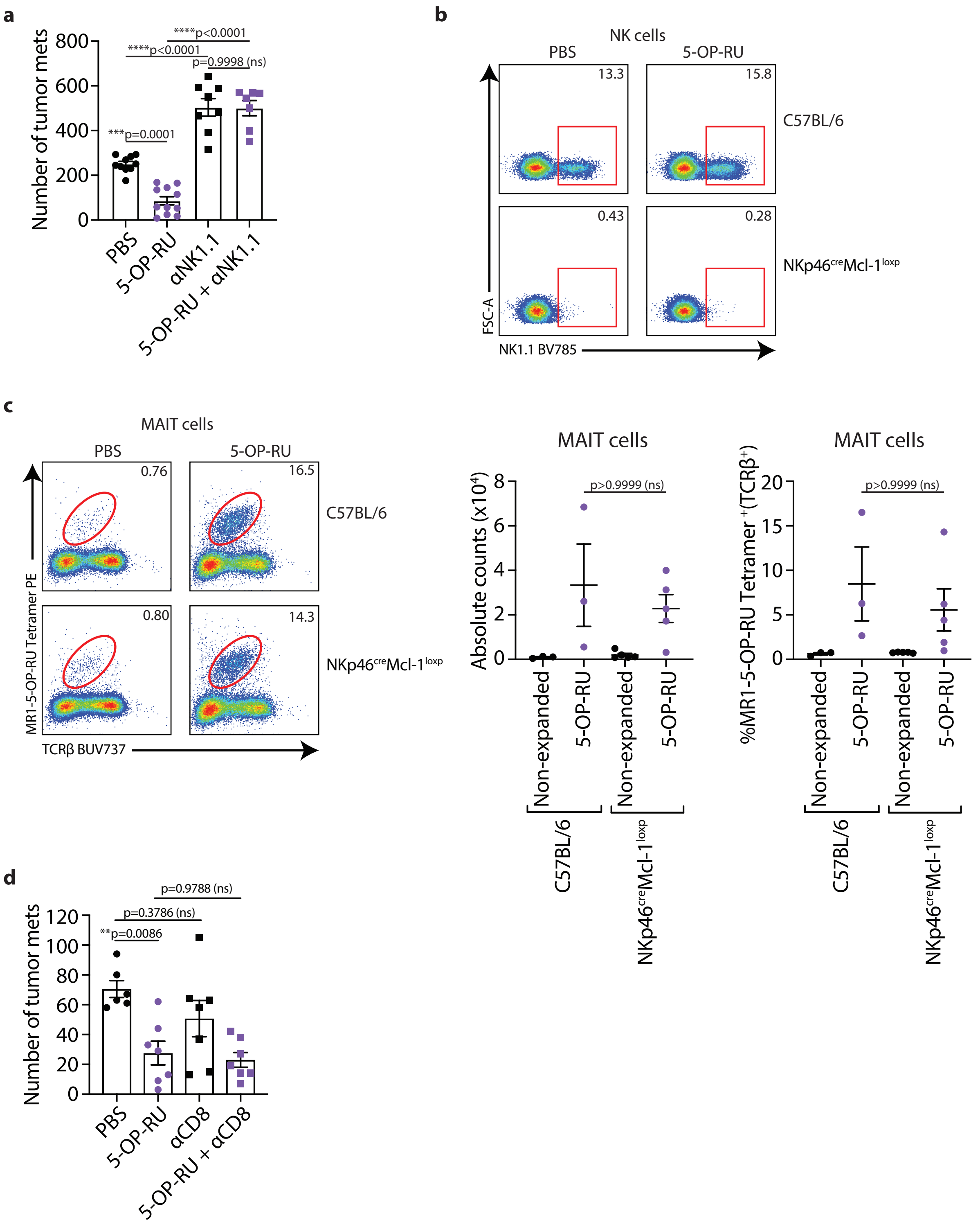

Supplementary Fig. 5 MAIT cells do not require NK cells for antigen-mediated expansion. (a) B6-MAIT$_{cast}$ MR1 WT mice were treated with PBS or 5-OP-RU as per **Figure 3a** and 2 x 10^5 B16F10 cells were injected i.v. on day 7. NK1.1$^+$ cells were targeted on days 4 and 7. Lungs were harvested and the number of tumor metastases enumerated 14 days after inoculation.Data presented as mean ± SEM of $n = 10$ (non-treated), $n = 11$ (5-OP-RU), $n = 8$ (αNK1.1) and n = 7 (5-OP-RU + αNK1.1) independent mice from one independent experiment, One-way ANOVA. **(b-c)** C57BL/6 and NKp46^{Cre}-Mcl-1l^{oxp} mice were treated with PBS or 5-OP-RU as per **Figure 3a**. At day 6 the numbers and proportion of NK cells (**b**) and MAIT cells (**c**) were determined by flow cytometry. Data presented as representative flow cytometry plots and mean ± SEM of $n = 3$ (C57BL/6) and $n = 5$ (NKp46^{Cre}-Mcl-1l^{oxp}) independent mice from one independent experiment, Kruskal-Wallis test. **(d)** C57BL/6 mice were treated as per **Figure 3a** and 2 x 10^5 B16F10 cells were injected i.v. on day 7. CD8$^+$ T cells were depleted on days 6 and 7. Lungs were harvested and the number of tumor metastases enumerated 14 days after inoculation. Data presented as mean ± SEM of $n = 6$ (PBS) and $n = 7$ (5-OP-RU, αCD8, 5-OP-RU + αCD8) independent mice from one independent experiment, One-way ANOVA. $^{**}p < 0.01$, $^{***}p < 0.001$, $^{****}p < 0.0001$, ns = non-significant.

Supplementary Figure 6

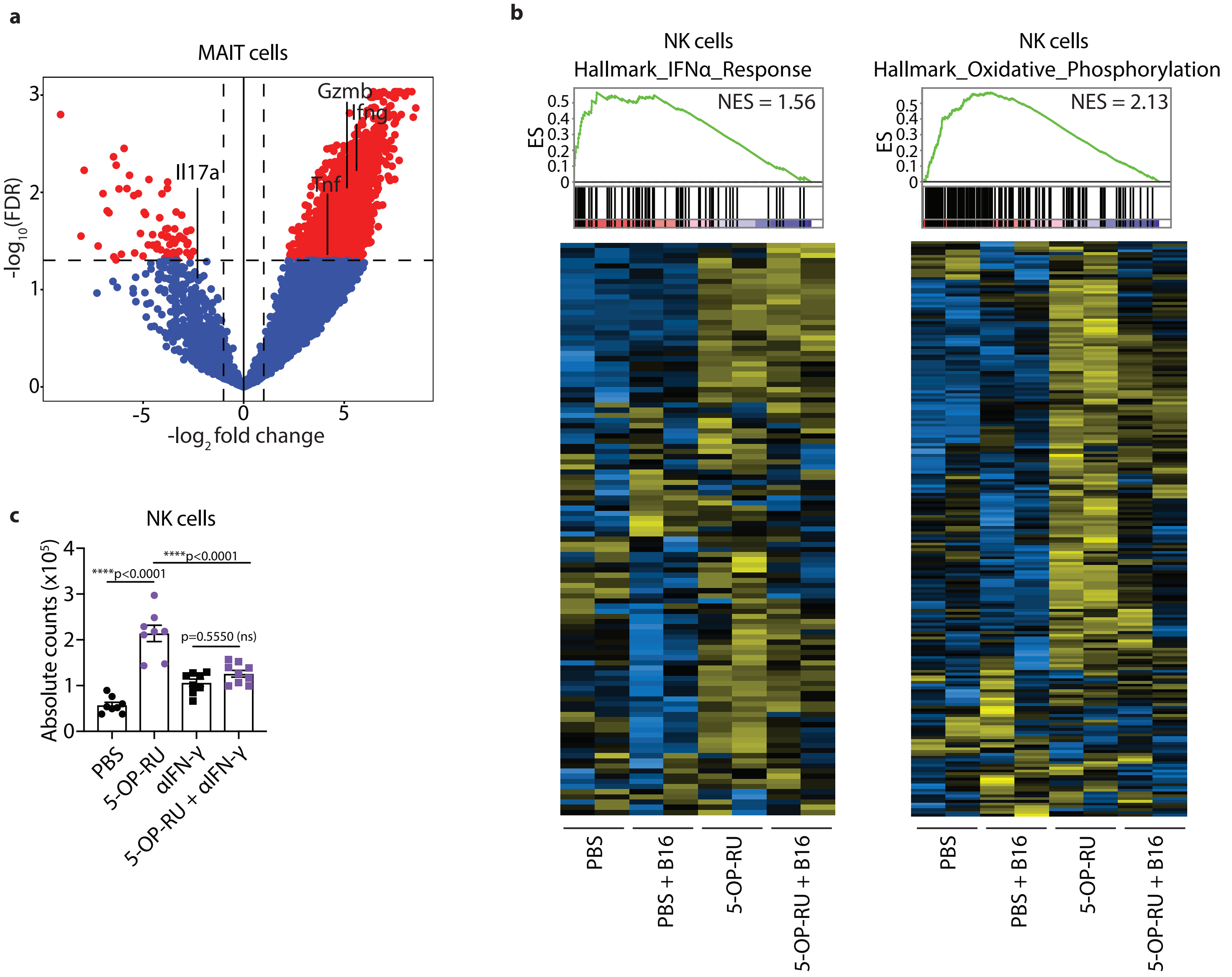

Supplementary Fig. 6 Gene expression changes in MAIT and NK cells following intranasal 5-OP-RU treatment. B6-MAIT$_{cast}$MR1 WT mice were treated with PBS or 5-OP-RU as per **Figure 3a.** 5 x 10^5 B16F10 melanoma cells, or PBS control, were injected i.v. at day 6 and 12 hours later lungs were processed. MAIT cells or NK1.1$^+$ NK cells were FACS sorted and RNA extracted for subsequent 3' RNA-sequencing analysis. Cells were harvested from three mice per group and data represented as technical duplicates. **(a)** Differential gene expression of MAIT cells treated with or without 5-OP-RU. **(b)** Heatmap showing differentially expressed genes from NK cells in hallmark IFNα response and hallmark oxidative phosphorylation pathways. **(c)** C57BL/6 mice were treated with PBS or 5-OP-RU as per **Figure 3a** and received 250 μg of αIFN-γ i.p. on day 0 and 1. Lungs were harvested at day 6 and absolute numbers of NK cells in the lungs determined. Data is presented as mean ± SEM of $n = 8$ (PBS, 5-OP-RU, αIFN-γ) and $n = 9$ (5-OP-RU + αIFN-γ) independent mice from one independent experiment, One-way ANOVA. ****$p < 0.0001$, ns = non-significant.

Supplementary Figure 7

a Gene list comprising MAIT signature

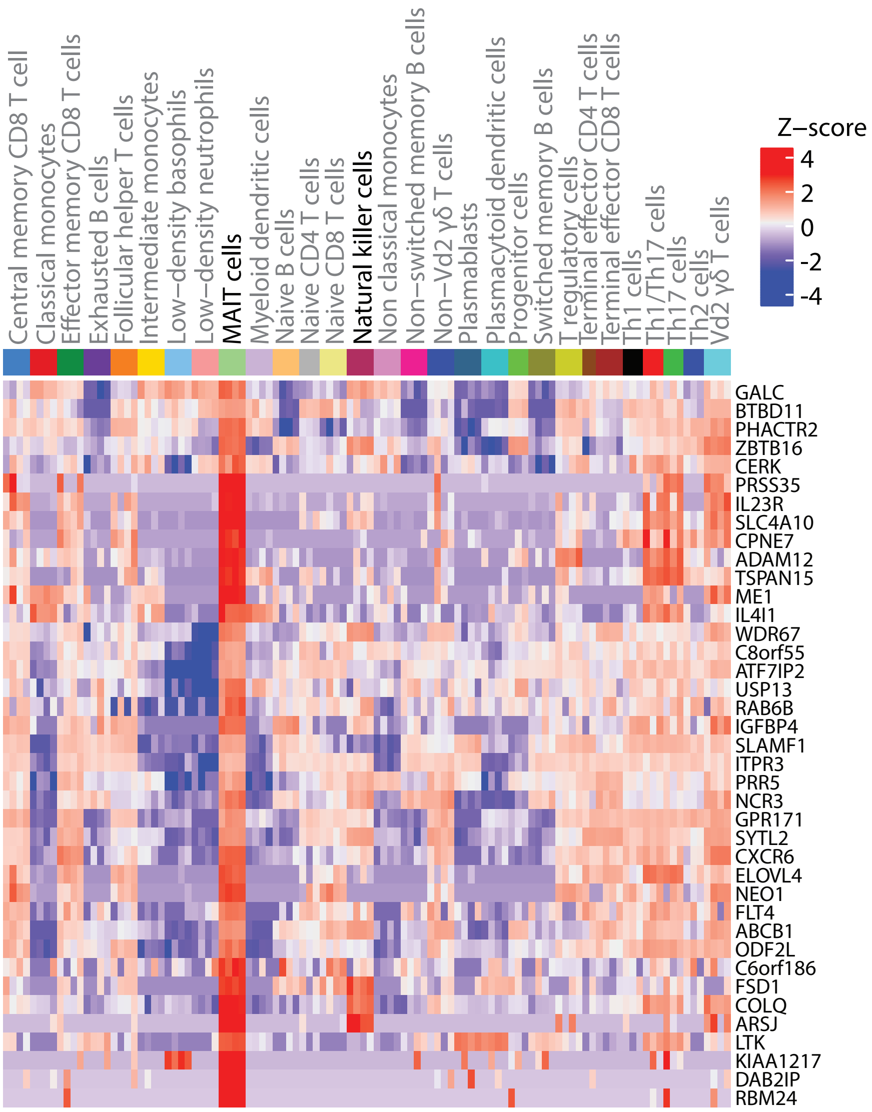

b Gene list comprising NK signature

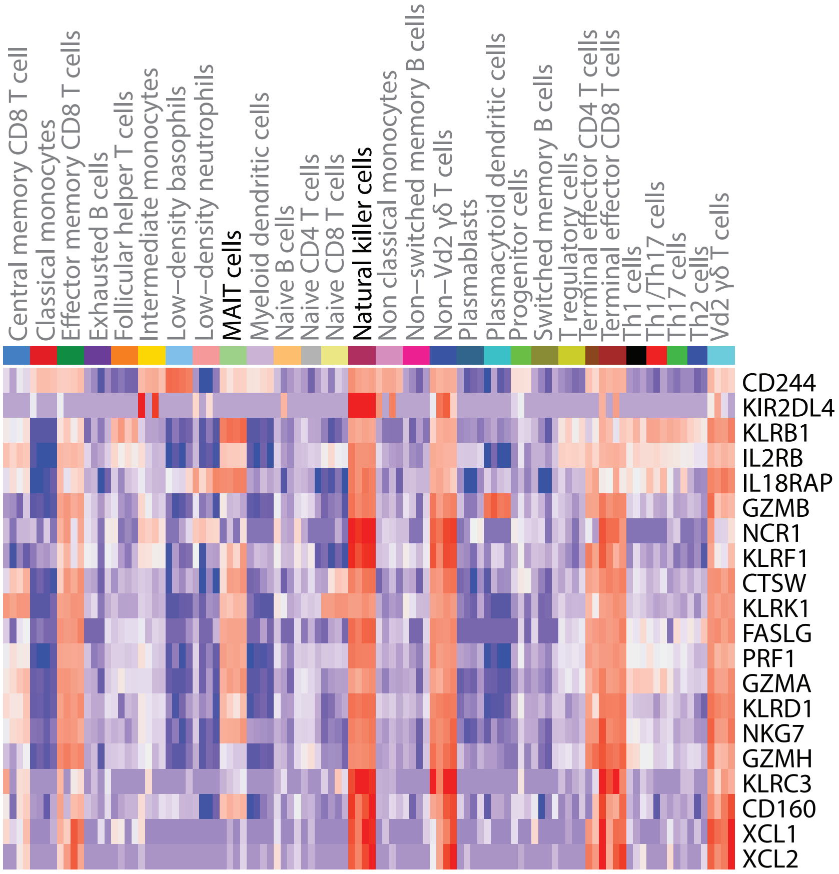

Supplementary Figure 7. Gene signature of MAIT cells and NK cells. Analysis of expression of genes associated with MAIT cells (**a**) and NK cells (**b**) in indicated immune cell types isolated from peripheral blood based on a published dataset *(43)*.

Supplementary Figure 8

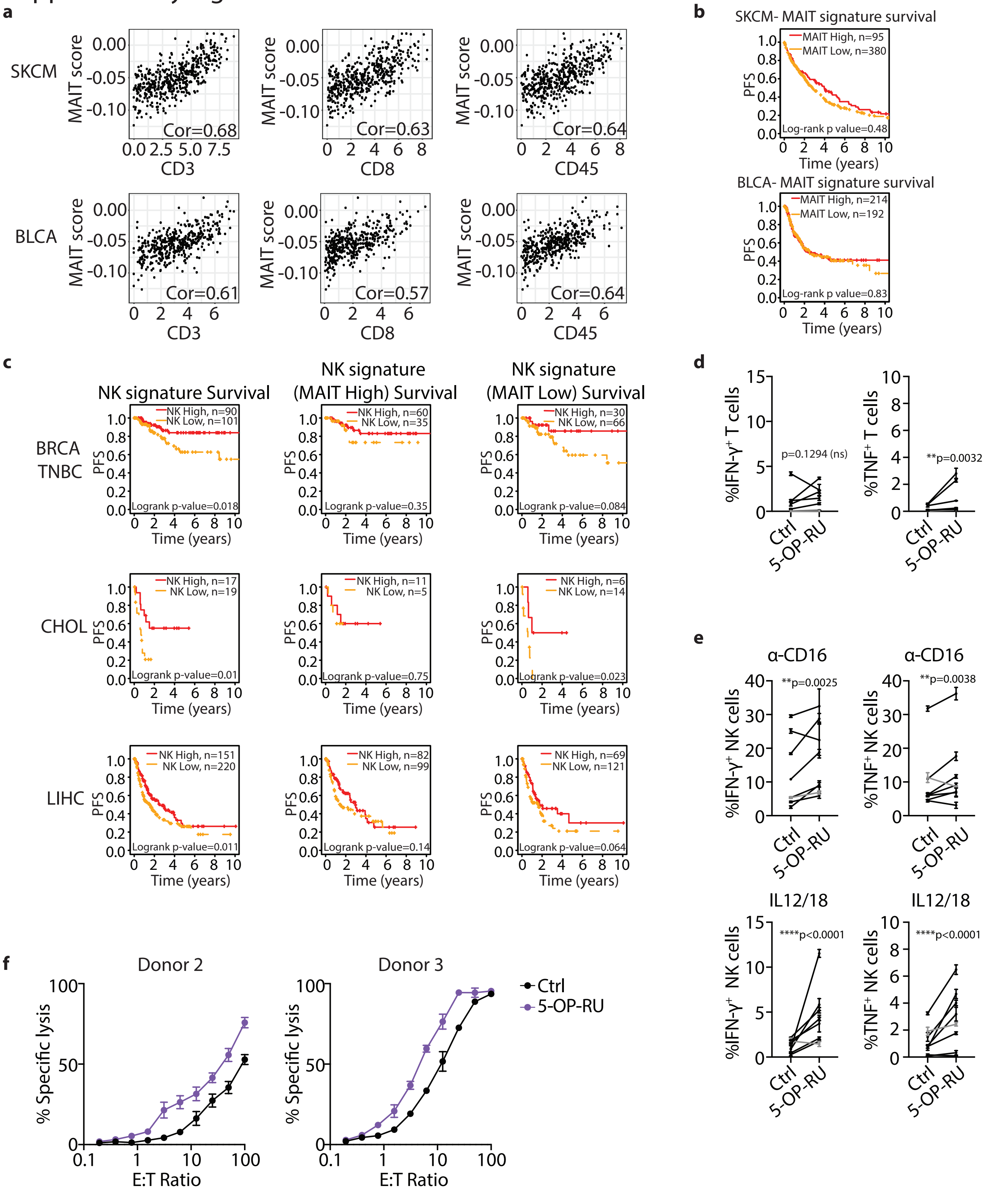

Supplementary Fig. 8 MAIT cell signature reduces the impact of a high NK cell score on progression-free survival. The impact of MAIT cells (MAIT signature) and NK cells (NK signature) on progression-free survival (PFS) of patients based upon TCGA datasets. **(a)** Correlation (cor) between MAIT cell score and expression of CD3, CD8 or CD45 as indicated for skin cutaneous melanoma (SKCM) and bladder carcinoma (BLCA) **(b)** Impact of MAIT cell signature score alone on patient PFS. **(c)** Impact of high and low MAIT cell signature score on PFS of patients with a high or low NK cell signature score as indicated for triple negative breast invasive carcinoma (BRCA TNBC), cholangiocarcinoma (CHOL) and liver hepatocellular carcinoma (LIHC). **(d)** PBMCs were cultured for 16 hours with IL-2 (20 IU/mL) in the presence or absence of 100 nM 5-OP-RU. Production of IFN-γ and TNF by $CD3^+$ T cells was determined by flow cytometry. Data is presented as mean ± SEM of triplicate cultures from eight independent donors, two-tailed Paired-t test. **(e)** PBMCs were cultured as in **d.** and stimulated with 10 μg/mL plate bound anti-CD16 or 500 pg/mL IL-12 and 500 pg/mL IL-18 for 4 hours at 37°C. Production of cytokines by $CD3^-CD56^+$ NK cells was determined by flow cytometry. Data is presented as mean ± SEM of triplicate cultures from eight independent donors, two-tailed Paired-t test. **F.** PBMCs were stimulated in IL-2 (20 IU/mL) with or without 100 nM 5-OP-RU and co-cultured with Cr^{51}-labelled K562 target cells as in **Figure 6e**. Data represented as the mean ± SEM of triplicate cultures. Individual replicates shown from donor 2 and donor 3. PBMC: tumor target ratios (E:T ratio) are as indicated. $^{**}p < 0.01$, $^{****}p < 0.0001$, ns = non-significant.

Supplementary Figure 9

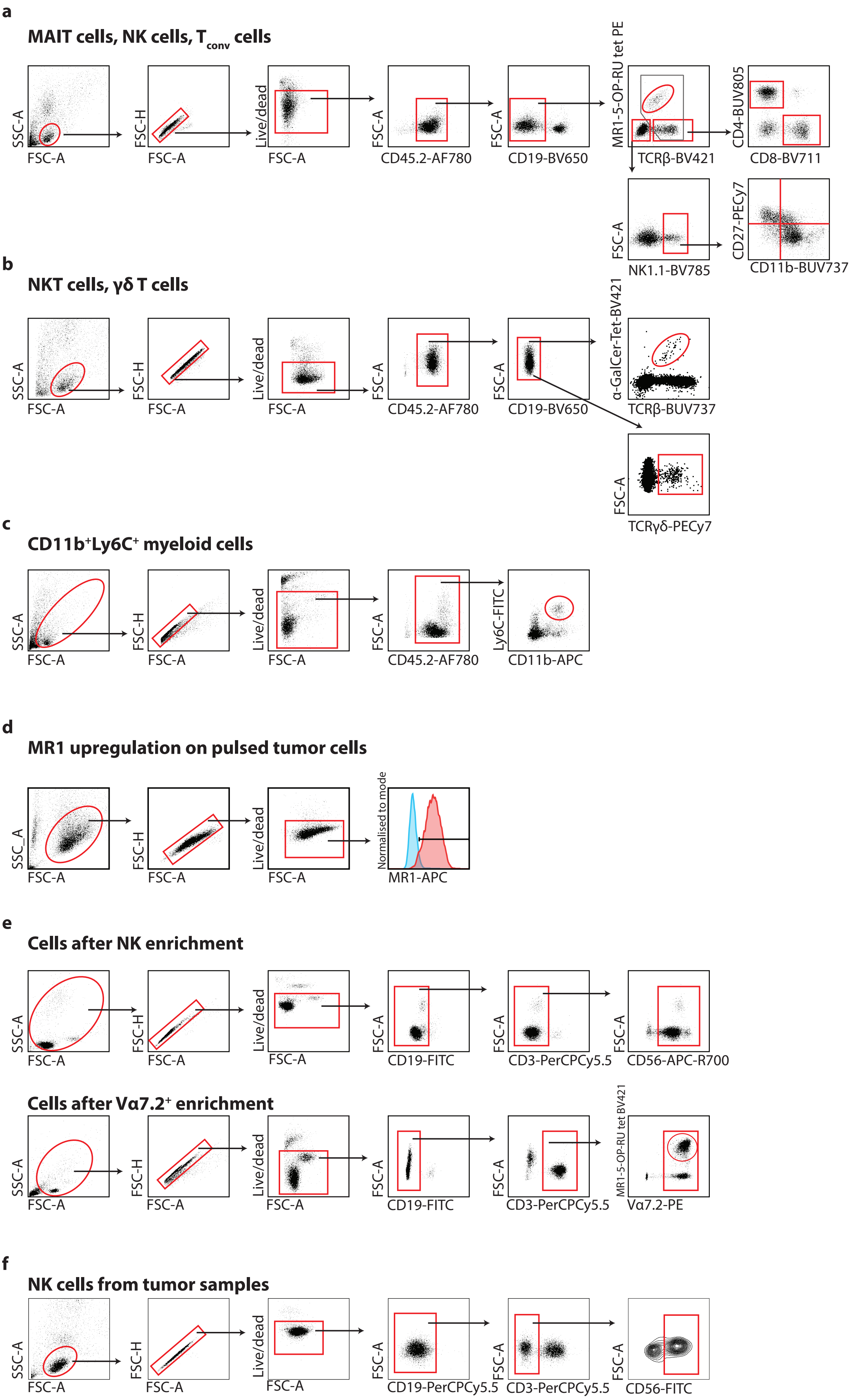

Supplementary Fig. 9 Gating strategies used for flow cytometry analysis. Cells of interest were gated on morphology via SSC-A and FSC-A. Doublets were excluded via FSC-H and FSC-A. Live cells were gated on. **(a)** Gating strategy for analyzing murine MAIT cells (CD45.2^{+}CD19^{-}TCRβ^{+} MR1-5OP-RU-tetramer^{+}), conventional T cells (CD45.2^{+}CD19^{-}TCRβ^{+}) then gated on either CD4^{+} or CD8^{+} cells and NK cells (CD45.2^{+}CD19^{-}TCRβ^{-}NK1.1^{+}) then gated on CD11b and CD27. **(b)** Gating strategy for analyzing murine NKT cells (CD45.2^{+}CD19^{-}TCRβ^{+}α-GalCer-tetramer^{+}) and γδ T cells (CD45.2^{+}CD19^{-}TCRγδ^{+}). **(c)** Gating strategy for analyzing murine CD11b^{+}Ly6C^{+} myeloid cells (CD45.2^{+}CD11b^{+}Ly6C^{+}). **(d)** Gating strategy for upregulation of MR1 on tumor cells. **(e)** Gating strategy to analyze human NK cells (CD19^{-}CD3^{-}CD56^{+}) and Vα7.2 cells (CD19^{-}CD3^{+}Vα7.2^{+}MR1-5OP-RU tetramer^{+}) after bead sort. (**f**) Gating strategy to analyse for NK cells (CD19^{-}CD3^{-}CD56^{+}) from tumor samples

Supplementary Table 1

Probe name	Fold change
**C3aR1**	8.32 +/- 3.23
**Serpinb2**	7.66 +/- 3.13
**Rorc**	5.87 +/- 3.74
**Cxcr6**	5.79 +/- 3.07
**C1qb**	5.31 +/- 3.46
**Tnfsf11**	4.40 +/- 1.67
**Ccl17**	4.01 +/- 1.26
**Cxcr3**	3.94 +/- 0.98
**C1qa**	3.82 +/- 2.47
**Chil3**	3.77 +/- 1.41
**Ccl9**	3.74 +/- 1.79
**Tnfsf14**	3.73 +/- 1.24
**Il1rl1**	3.64 +/- 0.58
**Prg2**	3.59 +/- 0.97
**Cxcl16**	3.39 +/- 1.30
**Icos**	3.22 +/- 1.06
**Trem2**	3.16 +/- 1.18
**Slc7a11**	2.79 +/- 1.25
**Ctla4**	2.79 +/- 0.64
**Fcgr2b**	2.76 +/- 0.96

Supplementary Table 1. Genes increased with 5-OP-RU treatment
List of top 20 genes expressed on lungs 5 days post 5-OP-RU treatment compared to PBS treated lungs, ranked by fold change.

Supplementary Table 2

MAIT cell signature genes list		
ZBTB16	GALC	TSPAN15
COLQ	WDR67	PRSS35
LTK	RBM24	ARSJ
ADAM12	SLAMF1	CERK
CXCR6	IGFBP4	IL4I1
ME1	ODF2L	PRR5
PHACTR2	NEO1	USP13
SYTL2	CPNE7	ITPR3
SLC4A10	NCR3	ATF7IP2
RORA	IL23R	RAB6B
RORC	FLT4	FSD1
DAB2IP	GPR171	C8orf55
KLRB1	ELOVL4	C6orf186
KIAA1217	ABCB1	BTBD11

Supplementary Table 2. List of MAIT cell signature genes
List of MAIT gene signature devised from differential genes expressed in MAIT cells compared to 28 other immune cell types *(43)*.

Supplementary Table 3

Reagent	Source	Catalogue	Dilution factor	Lot number
**Anti-mouse CD226, Clone TX42.1**	BioLegend	133615	1:100	B242082
**Anti-mouse TIGIT, Clone 1G9**	BioLegend	142106	1:100	B239608
**Anti-mouse CD96, Clone 3.3**	BioLegend	131712	1:100	B289664
**Anti-mouse CD314, Clone CX5**	eBioscience	25-5882-82	1:100	4337649
**Anti-mouse CD335, Clone 9E2**	BioLegend	331927	1:100	B256515
**Anti-human/mouse/rat MR1, Clone 26.5**	BioLegend	361106	1:200	B194444
**Mouse IgG2a κ, Clone MOPC-173**	BioLegend	400214	1:200	B213581
**Anti-mouse TCR γ/δ, Clone GL3**	BioLegend	118123	1:100	B198375
**Anti-mouse TCRβ, Clone H57-597**	BD Biosciences	612821	1:400	9063660
**Anti-mouse NK 1.1, Clone PK136**	eBioscience	11-5941-82	1:200	4306757
**Anti-mouse CD4, Clone GK1.5**	BD Biosciences	612900	1:400	9004535
**Anti-mouse CD49b, Clone DX5**	eBioscience	17-5971-82	1:200	E07352-1631
**Anti-mouse F4/80, Clone BM8**	Biolegend	123106	1:200	B253458
**Anti-mouse CD8a, Clone 53-6.7**	Biolegend	100748	1:400	B283363
**Anti-mouse B220/CD45R, Clone RA3-6B2**	Biolegend	103206	1:400	B247731
**Anti-mouse CD11c, Clone N418**	Biolegend	117336	1:200	B265353
**Anti-mouse CD45.2, Clone 104**	eBioscience	47-0454-82	1:200	7292830
**Anti-mouse CD64, Clone X54-5/7.1**	Biolegend	139306	1:100	B277148
**Anti-mouse MHC II, I-A/I-E Clone M5/114.15.2**	Biolegend	107622	1:200	B264454
**Anti-mouse Ly6G, Clone 1A8**	BD Biosciences	551460	1:400	1960
**Anti-mouse Ly6C, Clone HK1.4**	Biolegend	128018	1:400	B247616
**Anti-mouse Thy1.2, Clone 53-1.2**	Biolegend	140304	1:400	14477
**Anti-mouse CD103, Clone 2E7**	Biolegend	121406	1:100	B282445
**Anti-mouse CD19, Clone ID3**	BD Biosciences	553785	1:400	6320628
**Anti-mouse CD11b, Clone M1/70**	Biolegend	101242	1:400	B254686
**Anti-mouse CD69, Clone H1.2F3**	eBioscience	13-0691-82	1:100	3297541
**Anti-mouse TNF, Clone MP6-XT22**	Biolegend	506328	1:200	B245201
**Anti-mouse PD-1, Clone 29F.1A12**	Biolegend	135214	1:100	B277058
**Anti-mouse IFN-γ, Clone XMG1.2**	Biolegend	505808	1:200	B278613
**Viability Fixable Yellow**	Invitrogen	L34968	1:400	2009710
**Anti-mouse CD27, Clone LG 3A10**	BD Biosciences	560691	1:100	8135899
**Anti-mouse/human Ki67, Clone B56**	BD Biosciences	561126	1:100	8297779
**Anti-mouse KLRG1, Clone 2F1/KLRG1**	Biolegend	138411	1:100	B246929
**Anti-mouse EOMES, Clone Dan11mag**	Invitrogen	25-4875-82	1:200	4332806
**Anti-mouse CD107a, Clone 1D4B**	BD Pharmigen	558661	1:400	3242645
**Anti-human CD3, Clone UCHT1**	BioLegend	300430	1:100	B295216
**Anti-human CD8, Clone SK1**	BD Biosciences	612754	1:400	9164571
**Anti-human CD4, Clone SK3**	BD Biosciences	612887	1:200	237299
**Anti-human CD161, Clone HP-3G10**	BioLegend	339928	1:100	B289164
**Anti-human TCR Vα7.2, Clone 3C10**	BioLegend	351732	1:100	B306627
**Anti-human TIGIT, Clone A15153G**	BioLegend	372722	1:100	B237354
**Anti-human CD96, Clone NK92.39**	BioLegend	338417	1:100	B245661
**Anti-human CD335, Clone 9E2**	BioLegend	331927	1:100	B270439
**Anti-human CD16, Clone 3G8**	BioLegend	302046	1:200	B243830
**Anti-human CD69, Clone FN50**	BioLegend	310912	1:200	B259693
**Anti-human CD56, Clone NCAM16.2**	BD Biosciences	657886	1:100	8219679
**Anti-human CD19, Clone 4G7**	BD Biosciences	347543	1:100	62257
**Anti-human CD107a, Clone eBioH4A3**	eBioscience	11-1079-42	1:200	1975230
**Anti-human TCRαβ, Clone IP26**	BD Biosciences	564728	1:100	8354998

Supplementary Table 3 cont.

Reagent	Source	Catalogue	Dilution factor	Lot number
**Anti-human IFN-γ, Clone 4S.B3**	BD Biosciences	557844	1:200	156463
**Anti-human TNFα, Clone Mab11**	eBioscience	17-7349-82	1:200	4314341
**Anti-human CD314, Clone 1D11**	eBioscience	25-5878-41	1:100	4304097

Supplementary Table 3. List of antibodies used for flow cytometry.
List of mouse and human antibodies, source, catalogue number, dilution factor used for staining and lot number.
