# Supplementary material for: MAIT cells regulate NK cell-mediated tumor immunity
Source: Nat Commun. 2021 Aug 6;12:4746. doi: 10.1038/s41467-021-25009-4 (PMC8346465; doi:10.1038/s41467-021-25009-4)
Supplement: Supplementary file 3 — Reporting Summary [file 41467_2021_25009_MOESM3_ESM.pdf]

nature research

Corresponding author(s): Prof. Phil Darcy, Dr. Paul Beavis, Prof. Dale Godfrey

Last updated by author(s): Jul 13, 2021

Reporting Summary

Nature Research wishes to improve the reproducibility of the work that we publish. This form provides structure for consistency and transparency in reporting. For further information on Nature Research policies, see our Editorial Policies and the Editorial Policy Checklist.

Statistics

For all statistical analyses, confirm that the following items are present in the figure legend, table legend, main text, or Methods section.

n/a	Confirmed	
☐	☒	The exact sample size (*n*) for each experimental group/condition, given as a discrete number and unit of measurement
☐	☒	A statement on whether measurements were taken from distinct samples or whether the same sample was measured repeatedly
☐	☒	The statistical test(s) used AND whether they are one- or two-sided<br>*Only common tests should be described solely by name; describe more complex techniques in the Methods section.*
☒	☐	A description of all covariates tested
☐	☒	A description of any assumptions or corrections, such as tests of normality and adjustment for multiple comparisons
☐	☒	A full description of the statistical parameters including central tendency (e.g. means) or other basic estimates (e.g. regression coefficient) AND variation (e.g. standard deviation) or associated estimates of uncertainty (e.g. confidence intervals)
☐	☒	For null hypothesis testing, the test statistic (e.g. *F*, *t*, *r*) with confidence intervals, effect sizes, degrees of freedom and *P* value noted<br>*Give P values as exact values whenever suitable.*
☒	☐	For Bayesian analysis, information on the choice of priors and Markov chain Monte Carlo settings
☒	☐	For hierarchical and complex designs, identification of the appropriate level for tests and full reporting of outcomes
☒	☐	Estimates of effect sizes (e.g. Cohen's *d*, Pearson's *r*), indicating how they were calculated

Our web collection on statistics for biologists contains articles on many of the points above.

Software and code

Policy information about availability of computer code

Data collection	Flow cytometry: BD FacsDiva version 8 software (Flowjo LLC)<br>RNA-sequencing: NextSeq (Illumina)
Data analysis	Flow cytometry: FlowJo (version 10.2)<br>Statistical analysis and data presentation: Graphpad Prism (versions 7 and 8)<br>NanoString data: nSolver 4.0<br>Gene set enrichment analysis: Enrichr (http://amp.pharm.mssm.edu/Enrichr)<br>RNA sequencing: CASAVA v1.8.2, RNA-SeQC v1.1.7, Cutadapt v1.6, subread software package v1.6.4, Ensembl Release v96, Limma R and Voom v3.13, pheatmap R package v1.0.12, GSEA v3.0 as described in methods

For manuscripts utilizing custom algorithms or software that are central to the research but not yet described in published literature, software must be made available to editors and reviewers. We strongly encourage code deposition in a community repository (e.g. GitHub). See the Nature Research guidelines for submitting code & software for further information.

Data

Policy information about availability of data

All manuscripts must include a data availability statement. This statement should provide the following information, where applicable:

- Accession codes, unique identifiers, or web links for publicly available datasets
- A list of figures that have associated raw data
- A description of any restrictions on data availability

Data availability statement was not included in the submitted manuscript, but can be provided upon request. Accession codes will be provided if the manuscript is

accepted. Raw data for figure 4A, figure 5A-B, 5E, Supp figure 4A-C, Supp figure 6A-B can be provided to reviewers upon request.

Field-specific reporting

Please select the one below that is the best fit for your research. If you are not sure, read the appropriate sections before making your selection.

- [x] Life sciences
- [ ] Behavioural & social sciences
- [ ] Ecological, evolutionary & environmental sciences

For a reference copy of the document with all sections, see nature.com/documents/nr-reporting-summary-flat.pdf

Life sciences study design

All studies must disclose on these points even when the disclosure is negative.

Sample size	Experiments were performed with sufficient power to achieve statistical significance. All experiments were performed with the number of mice indicated in the corresponding figure legend.
Data exclusions	No data were excluded from the manuscript
Replication	The majority of experiments were replicated 2-3 times. Refer to figure legends for more information.
Randomization	All groups were aged and sexed matched. Randomization was not required for our experimental set ups. For in vitro studies randomization was not required since a homogeneous pool of tumor cells and/or immune cells were used with the indicated conditions.
Blinding	The investigators performed, acquired and analyzed the experiments and as such were not blinded.

Reporting for specific materials, systems and methods

We require information from authors about some types of materials, experimental systems and methods used in many studies. Here, indicate whether each material, system or method listed is relevant to your study. If you are not sure if a list item applies to your research, read the appropriate section before selecting a response.

Materials & experimental systems

n/a	Involved in the study	
[ ]	[x]	Antibodies
[ ]	[x]	Eukaryotic cell lines
[x]	[ ]	Palaeontology and archaeology
[ ]	[x]	Animals and other organisms
[ ]	[x]	Human research participants
[x]	[ ]	Clinical data
[x]	[ ]	Dual use research of concern

Methods

n/a	Involved in the study	
[x]	[ ]	ChIP-seq
[ ]	[x]	Flow cytometry
[x]	[ ]	MRI-based neuroimaging

Antibodies

Antibodies used

All antibodies used in this study were obtained from commercial suppliers (BD Pharmingen, eBioscience, Invitrogen, Thermo Scientific, Biolegend BioXcell or Wako Chemicals). A list of relevant information on the antibodies (Supplier, Catalog number, clone, dilution, lot number, technical data or QC sheet) where available is provided in a Supplementary Table.

Anti-mouse CD226, Clone TX42.1 BioLegend 133615 1:100
Anti-mouse TIGIT, Clone 1G9 BioLegend 142106 1:100
Anti-mouse CD96, Clone 3.3 BioLegend 131712 1:100
Anti-mouse CD314, Clone CX5 eBioscience 25-5882-82 1:100
Anti-mouse CD335, Clone 9E2 BioLegend 331927 1:100
Anti-human/mouse/rat MR1, Clone 26.5 BioLegend 361106 1:200
Mouse IgG2a κ, Clone MOPC-173 BioLegend 400214 1:200
Anti-mouse TCR γ/δ, Clone GL3 BioLegend 118123 1:100
Anti-mouse TCRβ, Clone H57-597 BD Biosciences 612821 1:400
Anti-mouse NK 1.1, Clone PK136 eBioscience 11-5941-82 1:200
Anti-mouse CD4, Clone GK1.5 BD Biosciences 612900 1:400
Anti-mouse CD49b, Clone DX5 eBioscience 17-5971-82 1:200
Anti-mouse F4/80, Clone BM8 Biolegend 123106 1:200
Anti-mouse CD8a, Clone 53-6.7 Biolegend 100748 1:400
Anti-mouse B220/CD45R, Clone RA3-6B2 Biolegend 103206 1:400
Anti-mouse CD11c, Clone N418 Biolegend 117336 1:200

Anti-mouse CD45.2, Clone 104 eBioscience 47-0454-82 1:200
Anti-mouse CD64, Clone X54-5/7.1 Biolegend 139306 1:100
Anti-mouse MHC II, I-A/I-E Clone M5/114.15.2 Biolegend 107622 1:200
Anti-mouse Ly6G, Clone 1A8 BD Biosciences 551460 1:400
Anti-mouse Ly6C, Clone HK1.4 Biolegend 128018 1:400
Anti-mouse Thy1.2, Clone 53-1.2 Biolegend 140304 1:400
Anti-mouse CD103, Clone 2E7 Biolegend 121406 1:100
Anti-mouse CD19, Clone ID3 BD Biosciences 553785 1:400
Anti-mouse CD11b, Clone M1/70 Biolegend 101242 1:400
Anti-mouse CD69, Clone H1.2F3 eBioscience 13-0691-82 1:100
Anti-mouse TNF, Clone MP6-XT22 Biolegend 506328 1:200
Anti-mouse PD-1, Clone 29F.1A12 Biolegend 135214 1:100
Anti-mouse IFN-γ, Clone XMG1.2 Biolegend 505808 1:200
Viability Fixable Yellow Invitrogen L34968 1:400
Anti-mouse CD27, Clone LG 3A10 BD Biosciences 560691 1:100
Anti-mouse/human Ki67, Clone B56 BD Biosciences 561126 1:100
Anti-mouse KLRG1, Clone 2F1/KLRG1 Biolegend 138411 1:100
Anti-mouse EOMES, Clone Dan11mag Invitrogen 25-4875-82 1:200
Anti-mouse CD107a, Clone 1D4B BD Pharmigen 558661 1:400
Anti-human CD3, Clone UCHT1 BioLegend 300430 1:100
Anti-human CD8, Clone SK1 BD Biosciences 612754 1:400
Anti-human CD4, Clone SK3 BD Biosciences 612887 1:200
Anti-human CD161, Clone HP-3G10 BioLegend 339928 1:100
Anti-human TCR Vα7.2, Clone 3C10 BioLegend 351732 1:100
Anti-human TIGIT, Clone A15153G BioLegend 372722 1:100
Anti-human CD96, Clone NK92.39 BioLegend 338417 1:100
Anti-human CD335, Clone 9E2 BioLegend 331927 1:100
Anti-human CD16, Clone 3G8 BioLegend 302046 1:200
Anti-human CD69, Clone FN50 BioLegend 310912 1:200
Anti-human CD56, Clone NCAM16.2 BD Biosciences 657886 1:100
Anti-human CD19, Clone 4G7 BD Biosciences 347543 1:100
Anti-human CD107a, Clone eBioH4A3 eBioscience 11-1079-42 1:200
Anti-human TCRαβ, Clone IP26 BD Biosciences 564728 1:100
Anti-human IFN-γ, Clone 4S.B3 BD Biosciences 557844 1:200
Anti-human TNFα, Clone Mab11 eBioscience 17-7349-82 1:200
Anti-human CD314, Clone 1D11 eBioscience 25-5878-41 1:100
Anti-mouse IFNγ, Clone H22, BioXcell BE0312
Anti-mouse NK1.1, Clone PK136, BioXcell BE0036
Anti-mouse CD8, Clone YTS, BioXcell BE0117
Anti-asialo GM-1, Wako Chemicals 986-10001
Anti-human CD16, Clone 3G8, Biolegend, 302014

Validation

Anti-mouse CD226, Clone TX42.1 BioLegend 133615 1:100. Each lot of this antibody is quality control tested by immunofluorescent staining with flow cytometric analysis. For flow cytometric staining, the suggested use of this reagent is ≤0.5 µg per million cells in 100 µl volume. It is recommended that the reagent be titrated for optimal performance for each application.

Anti-mouse TIGIT, Clone 1G9 BioLegend 142106 1:100. Each lot of this antibody is quality control tested by immunofluorescent staining with flow cytometric analysis. For flow cytometric staining, the suggested use of this reagent is ≤1.0 µg per million cells in 100 µl volume. It is recommended that the reagent be titrated for optimal performance for each application.

Anti-mouse CD96, Clone 3.3 BioLegend 131712 1:100. Each lot of this antibody is quality control tested by immunofluorescent staining with flow cytometric analysis. For flow cytometric staining, the suggested use of this reagent is ≤0.5 µg per million cells in 100 µl volume. It is recommended that the reagent be titrated for optimal performance for each application.

Anti-mouse CD314, Clone CX5 eBioscience 25-5882-82 1:100. This Antibody was verified by Cell treatment to ensure that the antibody binds to the antigen stated.

Anti-mouse CD335, Clone 9E2 BioLegend 331927 1:100. Each lot of this antibody is quality control tested by immunofluorescent staining with flow cytometric analysis. For flow cytometric staining, the suggested use of this reagent is ≤0.125 µg per million cells in 100 µl volume. It is recommended that the reagent be titrated for optimal performance for each application.

Anti-human/mouse/rat MR1, Clone 26.5 BioLegend 361106 1:200. Each lot of this antibody is quality control tested by immunofluorescent staining with flow cytometric analysis. For flow cytometric staining, the suggested use of this reagent is ≤0.25 µg per million cells in 100 µl volume. It is recommended that the reagent be titrated for optimal performance for each application.

Mouse IgG2a κ, Clone MOPC-173 BioLegend 400214 1:200. The MOPC-173 immunoglobulin has unknown specificity. The isotype of this antibody is mouse IgG2a, κ. This antibody was chosen as an isotype control after screening on a variety of resting, activated, live, and fixed human tissues.

Anti-mouse TCR γ/δ, Clone GL3 BioLegend 118123 1:100. Each lot of this antibody is quality control tested by immunofluorescent

staining with flow cytometric analysis. For flow cytometric staining, the suggested use of this reagent is ≤0.25 µg per million cells in 100 µl volume. It is recommended that the reagent be titrated for optimal performance for each application.

Anti-mouse TCRβ, Clone H57-597 BD Biosciences 612821 1:400. Two-color flow cytometric analysis of TCRβ expression on mouse splenocytes. Mouse splenic leucocytes were preincubated with Purified Rat Anti-Mouse CD16/CD32 antibody (Mouse BD Fc Block™) (Cat. No. 553141/553142). The cells were then stained with PE Rat Anti-Mouse CD4 (Cat. No. 553048/553049/561837) and PE Rat Anti-Mouse CD8a (Cat. No.553033/553032/561095) antibodies and either BD Horizon™ BUV737 Hamster IgG2, λ1 Isotype Control (Cat. No. 612822; Left Plot) or BD Horizon BUV737 Hamster Anti-Mouse TCR β Chain antibody (Cat. No. 612821; Right Plot) at 0.5 µg/test. BD Via-Probe™ Cell Viability 7-AAD Solution (Cat. No. 555815/555816) was added to cells right before analysis. The two-color flowcytometric dot plot showing the correlated expression of TCRβ (or Ig Isotype control staining) versus CD4 and CD8a was derived from gated events with the forward and side light-scatter characteristics of viable (7-AAD-negative) splenic leucocytes. Flow cytometry and data analysis were performed using a BD LSRFortessa™ Cell Analyzer System and FlowJo™ software.

Anti-mouse NK 1.1, Clone PK136 eBioscience 11-5941-82 1:200. Applications Tested: The PK136 antibody has been tested by flow cytometric analysis of C57Bl/6 mouse splenocytes and can be used at less than or equal to 0.5 µg per test. A test is defined as the amount (µg) of antibody that will stain a cell sample in a final volume of 100 µL. Cell number should be determined empirically but can range from 10^5 to 10^8 cells/test. It is recommended that the antibody be carefully titrated for optimal performance in the assay of interest.

Anti-mouse CD4, Clone GK1.5 BD Biosciences 612900 1:400. Two color flow cytometric analysis of CD4 expression on mouse splenocytes. Mouse splenic leucocytes were preincubated with Purified Rat Anti-Mouse CD16/32 antibody (Mouse Fc Block™)(Cat. No. 553141/553142). The cells were then stained with APC Hamster Anti-Mouse CD3e antibody (Cat. No. 553066/561826) and either BD Horizon™ BUV805 Rat IgG2b, κ Isotype Control (Cat. No. 612762; Left Plot) or BD Horizon™ BUV805 Rat Anti-Mouse CD4 antibody (Cat. No. 612900; Right Plot) at 0.25 µg/test. The two-color fluorescence contour plot showing the correlated expression of CD4 (or Ig Isotype control staining) versus CD3e were derived from gated events with the forward and side light scatter characteristic of viable splenic leucocytes. Flow cytometry and data analysis were performed using a BD LSRFortessa™ X-20 Cell Analyzer System and FlowJo™ software. Data shown on this Technical Data Sheet are not lot specific.

Anti-mouse CD49b, Clone DX5 eBioscience 17-5971-82 1:200. Applications Reported: The DX5 antibody has been reported for use in flow cytometric analysis. Applications Tested: The DX5 antibody has been tested by flow cytometric analysis of mouse splenocytes.

Anti-mouse F4/80, Clone BM8 Biolegend 123106 1:200. Each lot of this antibody is quality control tested by immunofluorescent staining with flow cytometric analysis. For flow cytometric staining, the suggested use of this reagent is ≤0.25 µg per million cells in 100 µl volume. It is recommended that the reagent be titrated for optimal performance for each application.

Anti-mouse CD8a, Clone 53-6.7 Biolegend 100748 1:400. Each lot of this antibody is quality control tested by immunofluorescent staining with flow cytometric analysis. For immunofluorescent staining using the µg size, the suggested use of this reagent is ≤0.5 µg per million cells in 100 µl volume. For immunofluorescent staining using µl sizes, the suggested use of this reagent is 5 µl per million cells in 100 µl staining volume or 5 µl per 100 µl of whole blood. It is recommended that the reagent be titrated for optimal performance for each application.

Anti-mouse B220/CD45R, Clone RA3-6B2 Biolegend 103206 1:400. Each lot of this antibody is quality control tested by immunofluorescent staining with flow cytometric analysis. For flow cytometric staining, the suggested use of this reagent is ≤1.0 µg per million cells in 100 µl volume. It is recommended that the reagent be titrated for optimal performance for each application.

Anti-mouse CD11c, Clone N418 Biolegend 117336 1:200. Each lot of this antibody is quality control tested by immunofluorescent staining with flow cytometric analysis. For flow cytometric staining using the µg size, the suggested use of this reagent is ≤0.25 µg per million cells in 100 µl volume. For flow cytometric staining using the µl size, the suggested use of this reagent is 5 µl per million cells in 100 µl staining volume or 5 µl per 100 µl of whole blood. It is recommended that the reagent be titrated for optimal performance for each application.

Anti-mouse CD45.2, Clone 104 eBioscience 47-0454-82 1:200. Staining of BALB/c splenocytes with Anti-Mouse CD3e FITC (Product # 11-0031-82) and 0.5 µg of Mouse IgG2a kappa Isotype Control APC-eFluor® 780 (Product # 47-4724) (left) or 0.5 µg of Anti-Mouse CD45-2 APC-eFluor® 780 (right). Total viable cells were used for analysis.

Anti-mouse CD64, Clone X54-5/7.1 Biolegend 139306 1:100. Each lot of this antibody is quality control tested by immunofluorescent staining with flow cytometric analysis. For flow cytometric staining, the suggested use of this reagent is ≤1.0 µg per million cells in 100 µl volume. It is recommended that the reagent be titrated for optimal performance for each application.

Anti-mouse MHC II, I-A/I-E Clone M5/114.15.2 Biolegend 107622 1:200. Each lot of this antibody is quality control tested by immunofluorescent staining with flow cytometric analysis. The suggested use of this reagent is ≤0.25 µg per million cells in 100 µl volume. It is highly recommended that the reagent be titrated for optimal performance for each application.

Anti-mouse Ly6G, Clone 1A8 BD Biosciences 551460 1:400. Flow cytometric analysis of expression of Ly-6G on bone-marrow leukocytes. BALB/c bone-marrow cell suspensions were stained with either FITC Rat Anti-Mouse Ly-6G (Cat. No. 551460/561105, dashed line histograms) or FITC Rat IgG2a, κ Isotype Control (Cat. No. 553929, shaded histograms), in the presence of Purified Rat Anti-Mouse CD16/CD32 (Mouse BD Fc Block™), (Cat. No. 553141/553142). Non-viable leukocytes were excluded by staining with propidium iodide, and leukocyte subsets were distinguished by their light scatter profiles. Left panel displays lymphoid and erythroid cells, and the right panel displays myeloid cells. Flow cytometry was performed on a FACSCalibur™.

Anti-mouse Ly6C, Clone HK1.4 Biolegend 128018 1:400.Each lot of this antibody is quality control tested by immunofluorescent staining with flow cytometric analysis. For flow cytometric staining, the suggested use of this reagent is ≤ 0.25 µg per 106 cells in 100 µl volume. It is recommended that the reagent be titrated for optimal performance for each application.

Anti-mouse Thy1.2, Clone 53-1.2 Biolegend 140304 1:400. Each lot of this antibody is quality control tested by immunofluorescent staining with flow cytometric analysis. For flow cytometric staining, the suggested use of this reagent is ≤0.25 µg per million cells in 100 µl volume. It is recommended that the reagent be titrated for optimal performance for each application.

Anti-mouse CD103, Clone 2E7 Biolegend 121406 1:100. Each lot of this antibody is quality control tested by immunofluorescent staining with flow cytometric analysis. For flow cytometric staining, the suggested use of this reagent is ≤0.25 µg per million cells in 100 µl volume. It is recommended that the reagent be titrated for optimal performance for each application.

Anti-mouse CD19, Clone ID3 BD Biosciences 553785 1:400. Two-color analysis of the expression of CD19 on mouse spleen B cells. BABL/c splenocytes were stained with PE-conjugated anti-mouse CD3e mAb 145-2C11 (Cat. No. 553063) in the absence (left panel) or presence (right panel) of FITC-conjugated mAb 1D3. Flow cytometry was performed on a BD FACScan™ flow cytometry system.

Anti-mouse CD11b, Clone M1/70 Biolegend 101242 1:400. Each lot of this antibody is quality control tested by immunofluorescent staining with flow cytometric analysis. For immunofluorescent staining using the µg size, the suggested use of this reagent is ≤0.4 µg per million cells in 100 µl volume. For immunofluorescent staining using the µl size, the suggested use of this reagent is 5 µl per million cells in 100 µl staining volume or 5 µl per 100 µl of whole blood. It is recommended that the reagent be titrated for optimal performance for each application.

Anti-mouse CD69, Clone H1.2F3 eBioscience 13-0691-82 1:100.Staining of ConA-stimulated BALB/c splenocytes with 0.25 µg of Armenian Hamster IgG Isotype Control Biotin (Product # 13-4888-81) (open histogram) or 0.25 µg of Anti-Mouse CD69 Biotin (filled histogram) followed by Streptavidin PE (Product # 12-4317-87). Total viable cells were used for analysis.

Anti-mouse TNF, Clone MP6-XT22 Biolegend 506328 1:200. Each lot of this antibody is quality control tested by intracellular immunofluorescent staining with flow cytometric analysis. For flow cytometric staining using the µg size, the suggested use of this reagent is ≤0.25 µg per million cells in 100 µl volume. For flow cytometric staining using the µl size, the suggested use of this reagent is 5 µl per million cells in 100 µl staining volume or 5 µl per 100 µl of whole blood. It is recommended that the reagent be titrated for optimal performance for each application.

Anti-mouse PD-1, Clone 29F.1A12 Biolegend 135214 1:100. Each lot of this antibody is quality control tested by immunofluorescent staining with flow cytometric analysis. For flow cytometric staining, the suggested use of this reagent is ≤1.0 µg per million cells in 100 µl volume. It is recommended that the reagent be titrated for optimal performance for each application.

Anti-mouse IFN-γ, Clone XMG1.2 Biolegend 505808 1:200.Each lot of this antibody is quality control tested by intracellular immunofluorescent staining with flow cytometric analysis. For flow cytometric staining, the suggested use of this reagent is ≤0.25 µg per million cells in 100 µl volume. It is recommended that the reagent be titrated for optimal performance for each application.

Viability Fixable Yellow Invitrogen L34968 1:400.This kit has been optimized and validated for use with a violet laser flow cytometer.

Anti-mouse CD27, Clone LG 3A10 BD Biosciences 560691 1:100.Flow cytometric analysis of CD27 on mouse splenocytes. Left Panel: Splenocytes from C57BL/6 mice were stained either with a APC Hamster IgG1, κ isotype control (shaded) or with the APC Hamster Anti-Mouse CD27 antibody (unshaded). Histograms were derived from gated events based on light scattering characteristics for lymphocytes. Middle and Right Panels: Splenocytes from C57BL/6 mice were stained with both a PE Hamster Anti-Mouse CD3e antibody (Cat.No. 553064) and either a APC Hamster IgG1, κ isotype control (middle panel) or the APC Hamster Anti-Mouse CD27 antibody (right panel). Dot plots were derived from gated events based on light scattering characteristics for lymphocytes. Flow cytometry was performed on a BD™ LSR II flow cytometry system.

Anti-mouse/human Ki67, Clone B56 BD Biosciences 561126 1:100. Flow cytometric analysis of Ki-67 expression by proliferating Jurkat and noncycling human peripheral blood mononuclear cells (PBMC). Jurkat and PBMC were fixed and permeabilized with 70% ice cold ethanol, washed, and stained with Alexa Fluor 647 Mouse Anti-Ki-67 antibody (Cat. No. 561126) according to the BD Biosciences support protocol, Flow Cytometry Staining Protocol for Detection of Ki-67. The cells were then RNase A (Sigma Cat. No. R-5500) treated and counterstained with Propidium Iodide Staining Solution (Cat. No. 556463) to stain DNA . Two-color flow cytometric dot plots showing the correlated expression patterns of Propidium Iodide (DNA) staining versus Ki-67 were derived from gated events with the forward and side light-scatter characteristics of intact Jurkat cells (Left Panel) or PBMC (Right Panel). Flow cytometry was performed using a BD LSR™ II flow cytometry system.

Anti-mouse KLRG1, Clone 2F1/KLRG1 Biolegend 138411 1:100. Each lot of this antibody is quality control tested by immunofluorescent staining with flow cytometric analysis. For flow cytometric staining, the suggested use of this reagent is ≤0.25 µg per million cells in 100 µl volume. It is recommended that the reagent be titrated for optimal performance for each application.

Anti-mouse EOMES, Clone Dan11mag Invitrogen 25-4875-82 1:200.Surface staining of C57BL/6 splenocytes with Anti-Mouse NK1-1 eFluor® 450 (Product # 48-5941-82) followed by intracellular staining with 0.25 µg of Rat IgG2a K Isotype Control PE (Product # 12-4321-80) (left) or 0.25 µg of Anti-Mouse EOMES PE (right) using the Foxp3/Transcription Factor Staining Buffer Set (Product # 00-5523-00) and protocol. Total viable cells were used for analysis.

Anti-mouse CD107a, Clone 1D4B BD Pharmigen 558661 1:400. Flow cytometric analysis of PE-conjugated anti-mouse CD107a on mouse splenocytes. Murine splenocytes were permeabilized with BD Cytofix/Cytoperm™ fixation/permeabilization buffer (Cat. No.554722) and then stained with either PE anti-CD107a (clone 1D4B, Cat. No. 558661, solid line) or a PE rat IgG2a isotype control (catalog number 553930, dashed line). Intracellular staining was analysed by flow cytometry performed on a BDvFACSCalibur™ System. The histograms were derived from the gated events based on light scattering characteristics of viable splenocytes.

Anti-human CD3, Clone UCHT1 BioLegend 300430 1:100.Each lot of this antibody is quality control tested by immunofluorescent staining with flow cytometric analysis. For flow cytometric staining, the suggested use of this reagent is 5 µl per million cells in 100 µl

staining volume or 5 µl per 100 µl of whole blood.

Anti-human CD8, Clone SK1 BD Biosciences 612754 1:400. Flow cytometric analysis was performed on whole blood stained with the indicated conjugated antibody. Laser excitation was at 405 nm, 488 nm, or 635 nm. Representative data analyzed with a BD FACS™ brand flowcytometer is shown in the following plots.

Anti-human CD4, Clone SK3 BD Biosciences 612887 1:200. Flow cytometric analysis of CD4 expression on human peripheral blood lymphocytes. Human whole blood was stained with either PE Mouse IgG1, κ Isotype Control (Cat. No. 554680; dashed line histogram) or PE Mouse Anti-Human CD4 antibody (Cat. No. 565999; solid line histogram). The erythrocytes were lysed with BD FACS™ Lysing Solution (Cat. No. 349202). The fluorescence histogram showing CD4 expression (or Ig Isotype control staining) was derived from gated events with the forward and side light-scatter characteristics of intact lymphocytes. Flow cytometric analysis was performed using a BD LSRFortessa™ Cell Analyzer System.

Anti-human CD161, Clone HP-3G10 BioLegend 339928 1:100. Each lot of this antibody is quality control tested by immunofluorescent staining with flow cytometric analysis. For flow cytometric staining, the suggested use of this reagent is 5 µl per million cells in 100 µl staining volume or 5 µl per 100 µl of whole blood.

Anti-human TCR Vα7.2, Clone 3C10 BioLegend 351732 1:100. Each lot of this antibody is quality control tested by immunofluorescent staining with flow cytometric analysis. For flow cytometric staining, the suggested use of this reagent is 5 µl per million cells in 100 µl staining volume or 5 µl per 100 µl of whole blood.

Anti-human TIGIT, Clone A15153G BioLegend 372722 1:100. Each lot of this antibody is quality control tested by immunofluorescent staining with flow cytometric analysis. For flow cytometric staining, the suggested use of this reagent is 5 µl per million cells in 100 µl staining volume or 5 µl per 100 µl of whole blood.

Anti-human CD96, Clone NK92.39 BioLegend 338417 1:100. Each lot of this antibody is quality control tested by immunofluorescent staining with flow cytometric analysis. For flow cytometric staining, the suggested use of this reagent is 5 µl per million cells in 100 µl staining volume or 5 µl per 100 µl of whole blood.

Anti-human CD335, Clone 9E2 BioLegend 331927 1:100.Each lot of this antibody is quality control tested by immunofluorescent staining with flow cytometric analysis. For flow cytometric staining, the suggested use of this reagent is ≤0.125 µg per million cells in 100 µl volume. It is recommended that the reagent be titrated for optimal performance for each application.

Anti-human CD16, Clone 3G8 BioLegend 302046 1:200. Each lot of this antibody is quality control tested by immunofluorescent staining with flow cytometric analysis. For flow cytometric staining, the suggested use of this reagent is 5 µl per million cells in 100 µl staining volume or 5 µl per 100 µl of whole blood.

Anti-human CD69, Clone FN50 BioLegend 310912 1:200.Each lot of this antibody is quality control tested by immunofluorescent staining with flow cytometric analysis. For flow cytometric staining, the suggested use of this reagent is 5 µl per million cells in 100 µl staining volume or 5 µl per 100 µl of whole blood.

Anti-human CD56, Clone NCAM16.2 BD Biosciences 657886 1:100. Two-color flow cytometric analysis of CD56 expression on human peripheral blood lymphocytes. Human whole blood was stained with APC Mouse Anti-Human CD16 antibody (Cat. No. 561248) and either BD Horizon™ BV510 Mouse IgG2b, κ Isotype Control (Cat. No. 563025; Left Panel) or BD Horizon™ BV510 Mouse Anti-Human CD56 antibody (Cat. No. 563041; Right Panel). The erythrocytes were lysed with BD Pharm Lyse™ Lysing Buffer (Cat. No. 555899). The two-color flow cytometric dot plots show the correlated expression patterns of CD16 versus CD56 (or Ig Isotype control staining) for gated events with the forward and side light-scatter characteristics of intact peripheral blood lymphocytes. Flow cytometric analysis was performed using a BD™ LSR II Flow Cytometer System.

Anti-human CD19, Clone 4G7 BD Biosciences 347543 1:100. Flow cytometric analysis was performed on PBMCs with scatter gates set on the lymphocyte fraction. Laser excitation was at 488 nm. Representative data analysed with a BD FACS™ brand flow cytometer is shown in the following figure.

Anti-human CD107a, Clone eBioH4A3 eBioscience 11-1079-42 1:200. Intracellular staining of the Jurkat cell line with 0.5 µg of Mouse IgG1 K Isotype Control FITC (Product # 11-4714-42) (open histogram) or 0.5 µg of Anti-Human CD107a (LAMP-1) FITC (filled histogram). Total cells were used for analysis.

Anti-human TCRαβ, Clone IP26 BD Biosciences 564728 1:100. Two-color Flow Cytometric Analysis TCRαβ Expression on Human Peripheral Blood Lymphocytes Left Panel - Whole blood was treated with BD Pharm Lyse™ Lysing Buffer (Cat. No. 555899) to lyse erythrocytes. The cells were washed and then stained with FITC Mouse Anti-Human CD19 antibody (Cat. No. 340409) and either PE Mouse IgG1, κ Isotype Control (Cat No. 554680, Left Plot) or PE Mouse Anti-Human TCRαβ antibody (Cat. No. 564728; Right Plot). Two-color flow cytometric contour plots showing the coexpression of CD19 vs TCRαβ (or Ig Isotype control staining) were derived from gated events with the forward and side light-scatter characteristics of viable lymphocytes.

Anti-human IFN-γ, Clone 4S.B3 BD Biosciences 557844 1:200. Expression of IFN-γ by stimulated human peripheral blood lymphocytes. Human PBMC were stimulated for 6 hours with PMA (50 ng/ml final concentration; Sigma) and calcium ionophore A23187 (500 ng/ml final concentration; Sigma) in the presence of GolgiStop™ (2 µM final concentration; Cat. No.554724). Stimulated cells were stained with PE Mouse anti-Human CD8 (Cat. No. 555367) and either PE-Cy™7 Mouse anti-Human IFN-γ (Cat. No. 557844/560741/561036, left panel) or PE-Cy™7 mouse IgG1 κ isotype control (Cat. No. 557646, right panel) by using the BD Pharmingen™ staining protocol. Dot plots were derived from gated events with the forward, and side light scatter characteristics of lymphocytes. The quadrant markers for the bivariate dot plots were set based on the autofluorescence and isotype controls.

Anti-human TNFα, Clone Mab11 eBioscience 17-7349-82 1:200. Normal human peripheral blood cells were unstimulated (left) or stimulated for 5 hours with Cell Stimulation Cocktail (plus protein transport inhibitors) (Product # 00-4975-03) (right). Cells were intracellularly stained with Anti-Human CD4 PerCP-eFluor® 710 (Product # 46-0047-42) and Anti-Human TNF alpha APC using the Intracellular Fixation & Permeabilization Buffer Set (Product # 88-8824-00) and protocol. Cells in the lymphocyte gate were used for analysis.

Anti-human CD314, Clone 1D11 eBioscience 25-5878-41 1:100. Applications Reported: The 1D11 antibody has been reported for use in flow cytometric analysis. Applications Tested: This 1D11 antibody has been tested by flow cytometric analysis of normal human peripheral blood cells.

Anti-mouse IFNγ, Clone H22, BioXcell BE0312. Western blot data shown below confirms that this clone binds to its target antigen.

Anti-mouse NK1.1, Clone PK136, BioXcell BE0036.Western blot data shown below confirms that this clone binds to its target antigen.

Anti-mouse CD8, Clone YTS, BioXcell BE0117. Western blot data shown below confirms that this clone binds to its target antigen
.
Anti-asialo GM-1, Wako Chemicals 986-10001. Spleen cells of BALB/c□ were treated with Anti Asialo GM1 plus guinea pig complement. Remaining NK activities were tested in vitro by using YAC-1 cells as target(●). Effector/target ratio was 50:1. ○ represents NK activities of BALB/c spleen cells treated with complement.

Anti-human CD16, Clone 3G8, Biolegend, 302014. Each lot of this antibody is quality control tested by immunofluorescent staining with flow cytometric analysis. For flow cytometric staining, the suggested use of this reagent is ≤2.0 µg per million cells in 100 µl volume or 100 µl of whole blood. It is recommended that the reagent be titrated for optimal performance for each application.

Eukaryotic cell lines

Policy information about cell lines

Cell line source(s)

The mouse melanoma B16F10 cell line and the human leukemia K562 cell line were obtained from ATCC. The mouse breast adenocarcinoma E0771 cell line was a kind gift from Prof. Robin Anderson (Olivia Newton-John Cancer Centre, Victoria, Australia), who generated this cell line. The

Authentication

Cell lines were not authenticated but were utilized within 10 passages of a master stock to ensure their accuracy.

Mycoplasma contamination

All cell lines were tested negative for mycoplasma contamination.

Commonly misidentified lines
(See ICLAC register)

The cells lines are not listed on the ICLAC database.

Animals and other organisms

Policy information about studies involving animals; ARRIVE guidelines recommended for reporting animal research

Laboratory animals

C57BL/6 mice, B6-MAITcast MR1 WT, B6-MAITcast MR1-/-, and NKp46Cre-Mcl-1loxp mice were utilized as indicated. Studies were performed in sex-matched and aged-matched mice between 6-12 weeks of age. Both female and male mice were used.

Housing conditions:
Light cycle- Sunrise/sunset mode, at 6:30am they fade on for 30 mins, full lights on at 7am and fade off at 7:30pm for 30 mins, dark at 8pm. 14 hours dark. 10 hours light.
Temperature- 20-21C
Humidity- 45-60%
Bedding material- Crushed corncob bedding, irradiated; changed fortnightly

Wild animals

This study did not involve wild animals.

Field-collected samples

No field colelction samples were used in this study.

Ethics oversight

Ethics oversight was performed by the Peter MacCallum Cancer Centre Animals Experimentation Ethics Committee (AEEC).

Note that full information on the approval of the study protocol must also be provided in the manuscript.

Human research participants

Policy information about studies involving human research participants

Population characteristics

Specimen: HM005
Specimen type: Axillary LND
Gender: M
Subtype: Cutaneous
Stage: IIIB
Molecular: NRAS Q61R

Description: Naive to immunotherapy
Case details: On adjuvant nivolumab, no recurrence.
Current status: No recurrence
Age: 79

Specimen: HM006
Specimen type: Axillary LND
Gender: M
Subtype: Cutaneous
Stage: IIIB
Molecular: BRAF V600E
Description: Naive to immunotherapy
Case details: On adjuvant dab/tram no recurrence.
Current status: No recurrence
Age: 36

Specimen: HM007
Specimen type: Axillary LND
Gender: M
Subtype: Cutaneous
Stage: IIIC
Molecular: BRAF V600K
Description: Nivo-refractory lesion (developed while on adjuvant Nivo).
Case details: On adjuvant nivo (+SLN), developed significant locoregional recurrence. REsected. Now on Dab/Tram (no recurrence).
Current status: No recurrence
Age: 35

Recruitment

Participants were recruited through surgeons, as surgery was indicated as a curative treatment for metastatic melanoma.

Ethics oversight

Melanoma Research Victoria HREC 07/38.

Note that full information on the approval of the study protocol must also be provided in the manuscript.

Flow Cytometry

Plots

Confirm that:

- [x] The axis labels state the marker and fluorochrome used (e.g. CD4-FITC).
- [x] The axis scales are clearly visible. Include numbers along axes only for bottom left plot of group (a 'group' is an analysis of identical markers).
- [x] All plots are contour plots with outliers or pseudocolor plots.
- [x] A numerical value for number of cells or percentage (with statistics) is provided.

Methodology

Sample preparation

Murine lung samples were excised and digested postmortem using a cocktail of 1 mg/mL collagenase type IV (Sigma-Aldrich) and 0.01 mg/mL DNAse (Sigma-Aldrich). After digestion at 37 degrees Celsius for 30 minutes, cells were passed through 70 micron filters twice. Spleens were processed into single cells through a 70 micron filter. Single cell suspensions were additionally lysed with ACK lysis buffer to remove red blood cells.
Peripheral blood mononuclear cells were isolated via standard density gradient using Ficoll-paque Plus (GE healthcare) and red blood cells were lysed with ACK lysis buffer.

Instrument

FACS data was obtained on a 5-laser FACS Symphony (BD Pharmingen) Model number R660964R4002

Software

Data was analyzed using FlowJo software (version 10.2)

Cell population abundance

For experiments involving FACS sorting, the purity of sorted cells was verified to be greater than 90%.

Gating strategy

To analyze cells a preliminary FSC/SSC gate was utilized to gate on the morphology of leukocytes. Subsequently, a single gate (FSC-A vs FSC-H) was used to exclude doublets followed by a viability gate (Fixable Yellow) to exclude dead cells. From this population, relevant gating strategies for each cell are described.

- [x] Tick this box to confirm that a figure exemplifying the gating strategy is provided in the Supplementary Information.
