# Supplementary material for: MAIT cells regulate NK cell-mediated tumor immunity
Source: Nat Commun. 2021 Aug 6;12:4746. doi: 10.1038/s41467-021-25009-4 (PMC8346465; doi:10.1038/s41467-021-25009-4)
Supplement: Supplementary file 4 — Description of Additional Supplementary Files [file 41467_2021_25009_MOESM4_ESM.pdf]

Description of Additional Supplementary Files

File name: Supplementary Data 1

A list of the antibody information and reagents used.
